# Robot-Assisted Versus Conventional Freehand Fluoroscopy-Guided Percutaneous Screw Fixation in Femoral Neck Fractures: A Systematic Review and Meta-analysis

**DOI:** 10.7759/cureus.24258

**Published:** 2022-04-18

**Authors:** Abdulrahman O Al-Naseem, Ahmed R Gonnah, Hussain Al-Ali, Abdulaziz O Al-Naseem, Irfan Siddique

**Affiliations:** 1 School of Medicine, University of Manchester, Manchester, GBR; 2 School of Medicine, University of Liverpool, Liverpool, GBR; 3 Orthopaedic Surgery Department, McGill University Health Centre, Quebec, CAN; 4 School of Medicine, University of Glasgow, Glasgow, GBR; 5 Department of Complex Spinal Surgery, Salford Royal National Health Services (NHS) Foundation Trust, Manchester, GBR

**Keywords:** robot assisted, fixation, percutaneous, fracture, femoral neck

## Abstract

Robotic-assisted navigation for percutaneous femoral neck fracture fixation is a new technology that has shown enhanced intraoperative and postoperative outcomes compared to the conventional freehand fluoroscopy-guided technique. The authors aim to compare robot-assisted femoral neck fracture fixation to conventional freehand fluoroscopy-guided repair. Electronic databases were searched, identifying all observational studies comparing outcomes of both groups. Using the Preferred Reporting Items for Systematic Reviews and Meta-Analyses (PRISMA) guidelines, a systematic review and meta-analysis were conducted. The primary outcomes included operative duration (minutes), intraoperative bleeding (mL), fluoroscopy exposure, and frequency of intraoperative drilling. The secondary outcomes included Harris scores, healing rate and time, screw accuracy, and postoperative complications. Seven observational studies were identified, enrolling 506 patients. There was a significant difference between the robot-assisted and conventional groups in terms of intraoperative blood loss (mean difference (MD) = -18.83, p ≤ 0.05), fluoroscopy exposure (MD = -1.81, p ≤ 0.05), and intraoperative drilling frequency (MD = -7.35, p < 0.05). There was no significant difference in operative duration between the groups (MD = -0.21, p = 0.66). Most secondary outcomes were improved in the robot-assisted group. Overall, robot-assisted fixation was superior in terms of safety and efficacy.

## Introduction and background

Fractures of the femoral neck (NOF) are a specific type of intracapsular hip fractures [1] that occur most commonly due to low-energy falls in the elderly [2]. They can also occur in the younger population (<60 years), accounting for 2%-11% of cases [3], secondary to high-energy trauma such as traffic accidents and falls from significant heights [4]. Risk factors vary in the different age groups presenting with this injury. For instance, NOF in the elderly are more common in postmenopausal Caucasian females who have reduced physical activity and osteoporosis [1,4]. Younger patients with chronic debilitating conditions including seizures, impaired balance, or osteopenia (secondary to malnutrition, hemiplegia, or medications) are also at a higher risk [4]. Additionally, smoking and alcoholism have been associated with low peak bone mass and hence increased risk of hip complications [4]. Fractures are evaluated using plain films and computed tomography (CT) scans and classified according to Garden and Pauwel classifications [1], which are used to determine the management. Surgery is the mainstay of management.

Operative methods include open reduction and internal fixation (ORIF) in younger patients with displaced fractures [1]. Interruption of the blood supply (medial circumflex arteries) to the femoral head is concerning in these patients as it can lead to avascular necrosis (AVN) [1]. Patients with displaced fractures usually present with vertically oriented Pauwel type 3 fractures due to trauma [1], where percutaneous sliding and cannulated hip screws can be more beneficial [1]. Non-displaced fractures are usually managed similarly and most commonly with cannulated hip screws, which reduces the risk of AVN and nonunion [1]. Patients with displaced hip fractures undergo hemi- or total hip replacement, depending on their activity status [1]. The most common site for hip fracture is the trochanteric region and is usually stabilized by osteosynthesis using the sliding compression hip screw [5].

Current techniques involve placing the cannulated screw into the femoral neck/head with the help of a guidewire [5]. The conventional approach involves inserting the guidewire into the femoral head/neck using mobile C-arm fluoroscopy to obtain posterior-anterior (PA) and lateral-to-medial views of the fractured hip [5]. The lack of 3D X-ray images incurs a depth perception problem [5]. Postoperative follow-up outcomes have shown a 10% failure rate due to difficulties in the initial insertion of the guidewire, leading to excessive drilling and bleeding, prolonged procedure and fluoroscopy exposure time, and an increased risk of infection [5]. Additionally, bone damage can arise, which can also have implications for postoperative recovery [5]. Furthermore, computer-assisted surgery was previously trialled in practice to provide 3D images of the path of the guidewire insertion; however, the absence of the robotic manipulator drill did not solve the problem of precise insertion and was associated with more complications [6].

Recently studies on minimally invasive robotic-assisted orthopedic surgery have shown better outcomes in the surgeries of the sacroiliac joint [7] and spinal [8] percutaneous screw placement compared to the freehand technique. A study by Al-Naseem et al. has shown robot-assisted fixation for posterior pelvic ring injuries to have better intraoperative outcomes [9]. The guidewire insertion stage is the only targeted stage, involving intraoperative calibration and trajectory planning [5]. Calibration is achieved via the fluoroscopy-based intraoperative registration scheme and X-ray photogrammetry to introduce artificial calibration features (fiducials) [5] at which the guidewire can be inserted safely with minimal complications and effectively by drilling at a maximal 0.88 mm reconstruction error [5]. The position and direction of the sleeve are checked using intraoperative anteroposterior (AP) and lateral fluoroscopic X-ray images, which are also used to examine screw positioning (parallelism and neck-width coverage), allowing for position adjustment in cases of bias [5]. The normal steps are then carried out by the surgeon. Postoperative management regimens are similar in both groups, including prophylactic antibiotics for 24 hours after surgery, repeat anteroposterior pelvic and lateral hip radiographs at regular intervals, and physical therapy, to allow for weight-bearing walking once the fracture fully healed with evidence on X-ray [5]. The application of robot-assisted surgery in practice has been evaluated in some studies and a meta-analysis reviewing spine fixation [7].

## Review

Methods

A systematic review and meta-analysis were conducted as per the Preferred Reporting Items for Systematic Reviews and Meta-Analyses (PRISMA) guidelines [10].

Eligibility Criteria

All randomized control trials and observational studies comparing robot-assisted percutaneous cannulated screw fixation with conventional freehand screw fixation for femoral neck fractures were included. Robot-assisted fixation was the intervention group of interest, and conventional freehand fixation was the comparator.

Primary Outcomes

The primary outcomes included operation duration (minutes), intraoperative bleeding (mL), and intraoperative fluoroscopy and intraoperative drilling frequency. Operation duration was defined as the total time in minutes from the start of the aseptic technique to suture incision. The total volume of blood in milliliters (mL) collected in the suctioning machine was used as a measure of intraoperative bleeding. Fluoroscopy was also assessed using time in seconds. The frequency of intraoperative drilling was defined as the number of guidewire insertions for screw pathway planning.

Secondary Outcomes

The secondary outcomes included the Harris score, fracture healing rate and time, screw accuracy, and postoperative complications. Screw accuracy included anterior-posterior and lateral parallelism, as well neck area held by screws. Complications such as femoral head penetration and avascular necrosis were summarized in a table.

Literature Search Strategy

Two authors (AN and AG) independently searched the following electronic databases: MEDLINE, Embase, and the Cochrane Central Register of Controlled Trials (CENTRAL). The last search was run on March 24, 2021. Thesaurus headings, search operators, and limits in each of the above databases were adapted accordingly. In addition, the World Health Organization International Clinical Trials Registry (http://apps. who.int/trial search/), ClinicalTrials.gov (http://clinical- trials.gov/), and ISRCTN Register (http://www.isrctn. com/) were searched for details of ongoing and unpublished studies. No language restrictions were applied. Medical Subject Headings (MeSH) terms including “robot,” “fixation,” “fracture,” and “femoral” were utilized. The bibliographic lists of articles of relevance were reviewed.

Selection of Studies

The titles and abstracts of articles identified from the literature searches were assessed independently by two authors (AN and AG). The full texts of relevant reports were retrieved, and those articles that met the eligibility criteria of our review were selected. Discrepancies in study selection were resolved by discussion between the authors.

Data Extraction and Management

An electronic data extraction spreadsheet was created according to Cochrane’s data collection form for intervention reviews. Pilot testing of the spreadsheet was carried out in randomly selected articles, and adjustments were made accordingly. The data extraction spreadsheet provided data on the study characteristics (author, publication year, publication journal, country of study, study design, study size, clinical condition of the study participants, intervention type, and comparison), baseline demographics of the included populations (age and gender), and primary and secondary outcome data. The results were obtained and recorded by two authors separately (HA and AZ). Disagreements were solved by discussion.

Data Synthesis

Data synthesis was conducted via the Review Manager 5.4 software. Data extracted were input into Review Manager by two authors independently (AN and AZ). The analysis involved was mainly based on the fixed-effect model. The random-effect model was only used when heterogeneity was high (greater than 75%). The results were reported in forest plots with 95% confidence intervals (CIs).

For dichotomous outcomes, the odds ratio (OR) was calculated between the two groups. The OR is the odds of an event in the robot-assisted group compared with the conventional group. An OR of less than 1 for avascular necrosis would favor the robot-assisted group, an OR of more than 1 would favor the conventional group, and an OR of 1 would favor neither group. An OR of more than 1 for fracture healing rate would favor the robot-assisted group, an OR of less than 1 would favor the conventional group, and an OR of 1 would favor neither group.

For continuous outcomes, the mean difference (MD) was calculated between the two groups. A positive MD for the Harris score and neck area held by screws would favor the robot-assisted group, a negative MD would favor the conventional group, and an MD of 0 would favor neither group. For all other outcomes, a negative MD would favor the robot-assisted group, a positive MD would favor the conventional group, and an MD of 0 would favor neither group.

Assessment of Heterogeneity

Heterogeneity among the studies was assessed using the Cochran Q test (χ2). Inconsistency was quantified by calculating I2 and interpreted using the following guide: 0%-25% may represent low heterogeneity, 25%-75% may represent moderate heterogeneity, and 75%-100% may represent high heterogeneity.

Results

Literature Search Results

**Figure 1:**
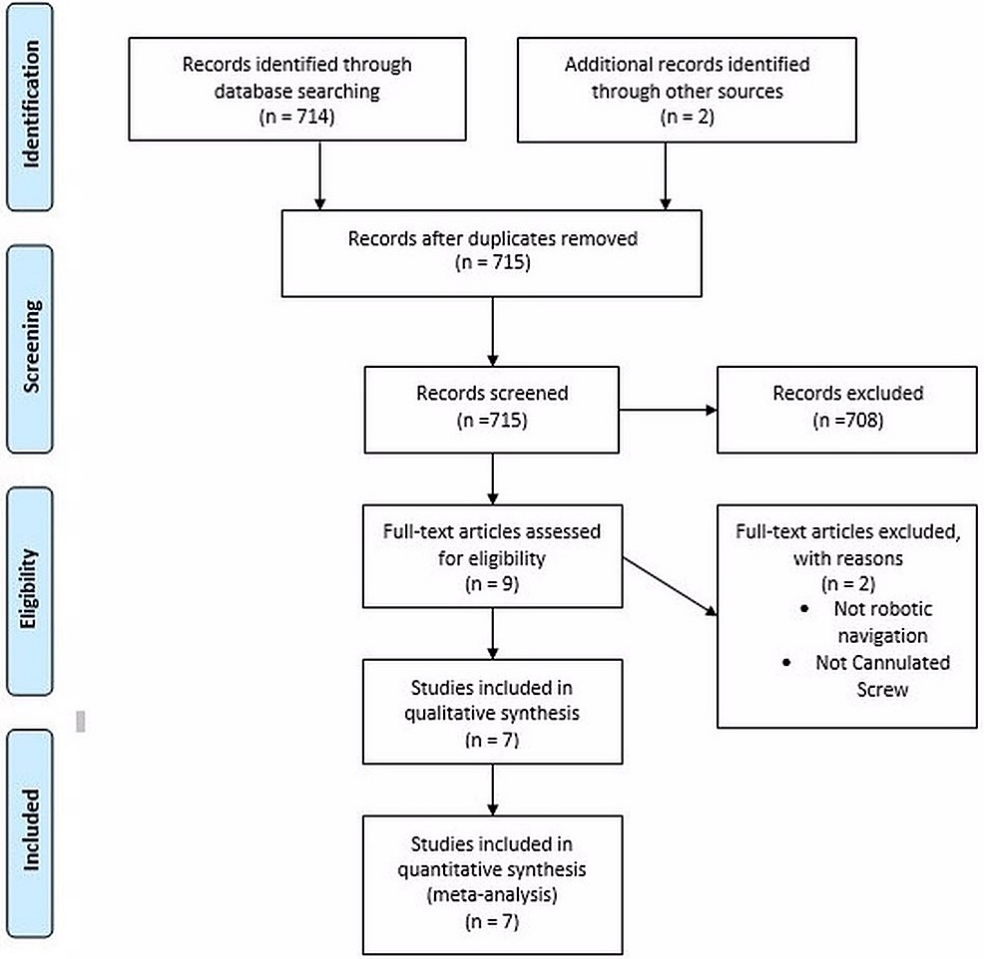
PRISMA flow diagram. The PRISMA diagram details the search and selection processes applied during the overview. PRISMA: Preferred Reporting Items for Systematic Reviews and Meta-Analyses

Figure 1 demonstrates our search strategy, which retrieved 716 studies. After a thorough screening process of the retrieved articles, the authors identified seven studies in total that met the eligibility criteria.

Description of the Included Studies

Seven studies were included, consisting of a total of 506 patients. Tong et al. (2016) [11] and Cao et al. (2017) [12] were the first two studies to look at robot-assisted femoral neck fixation. All studies except for Duan et al. (2019) [13], He et al. (2019) [14], and Wan et al. (2021) [15], which were prospective studies, were retrospective cohort studies. Duan et al. [13] was a prospective cohort study, while He et al. [14] and Wan et al. [15] were the only randomized control trials. Wang et al. (2019) [16] and Zhu et al. (2021) [17] were the largest studies with 128 and 133 patients each compared to the other studies that had a number of included patients in the range of 3-60. The studies aimed to assess the efficacy and safety of the included studies looking mainly at intraoperative outcomes. Reporting of complications has been noted to be poor among the studies. Further details can be found in Table 1.

**Table 1:** Baseline characteristics of the included studies. [11-17]

Author and year	Study design	Randomized	Single or multicenter	Intervention versus control	Patient population	Time from injury to operation (mean (SD))	Robot type	Mean age (SD)	Male to female ratio	Total number of study participants	Intervention group (robot-assisted)	Control group (freehand fluoroscopy-guided)	Follow-up time (months)
Tong et al. (2016) [11]	Retrospective cohort study	No	Single	Robot-assisted versus conventional freehand fluoroscopy	Femoral neck fracture	NA	TINAVI/TiRobot, Medical Technologies, Beijing, China	NA	NA	38	20	18	18 (mean)
Cao et al. (2017) [12]	Retrospective cohort study	No	Single	Robot-assisted versus conventional freehand fluoroscopy	Femoral neck fracture	NA	UR Positioning Robots, Universal Robots, Odense, Denmark	NA	NA	56	20	36	14.7 (mean)
He et al. (2019) [14]	Randomized controlled trial	Yes	Single	Robot-assisted versus conventional freehand fluoroscopy	Femoral neck fracture	NA	TINAVI/TiRobot, Medical Technologies, Beijing, China	56 (39-82) versus 56.2 (30-84)	11/19 versus 12/18	60	30	30	12-24 (range)
Duan et al. (2019) [13]	Prospective cohort	No	Single	Robot-assisted versus conventional freehand fluoroscopy	Femoral neck fracture	6.3 ± 2.3 versus 6.5± 2.4	TINAVI/TiRobot, Medical Technologies, Beijing, China	61.7 ± 5.2 versus 62.1 4.1	11/15 versus 9/14	49	26	23	13.6 (mean)
Wang et al. (2019) [16]	Retrospective cohort study	No	Single	Robot-assisted versus conventional freehand fluoroscopy	Femoral neck fracture	NA	TINAVI/TiRobot, Medical Technologies, Beijing, China	49.03 (8.23) versus 49.8 (7.68)	30/33 versus 31/34	128	63	65	12-24 (range)
Wan et al. (2021) [15]	Randomized controlled trial	Yes	Single	Robot-assisted versus conventional freehand fluoroscopy	Femoral neck fracture	NA	TINAVI/TiRobot, Medical Technologies, Beijing, China	51.86 (4.89) versus 51.33 (4.3)	12/9 versus 14/7	42	21	21	6
Zhu et al. (2021) [17]	Retrospective cohort study	No	Single	Robot-assisted versus conventional freehand fluoroscopy	Femoral neck fracture	5.3 ± 3.8 versus 6 ± 4	TINAVI/TiRobot, Medical Technologies, Beijing, China	47.9 ± 13.5 versus 47.7 ± 12.6	26/24 versus 47/36	133	50	83	At least 24

Primary Outcomes

Intraoperative outcomes including operative duration (minutes), intraoperative bleeding (mL), and frequency of intraoperative fluoroscopy and drilling were assessed. These outcomes are summarized in Table 2.

**Table 2:** Primary intraoperative outcomes including total operation duration, fluoroscopy exposure, and intraoperative bleeding and drilling frequency. N: number, NR: not reported, RAF: robot-assisted fixation, CFF: conventional freehand fixation

Study	Group	N	Total operation duration (minutes) (mean ± SD)	Fluoroscopy exposure (mean ± SD)	Intraoperative bleeding (mL) (mean ± SD)	Drilling frequency (N) (mean ± SD)
Tong et al. (2016) [11]	RAF	20	79.7 ± 15.7	14 ± 4.5	8 ± 3.4	4.8 ± 0.8
	CFF	20	74.1 ± 14.9	21 ± 5.4	10 ± 2.4	11.8 ± 2.4
Cao et al. (2017) [12]	RAF	36	90.1 ± 18.61	11.6 ± 3.15	19.25 ± 9.5	3.55 ± 0.89
	CFF	18	79.36 ± 15.74	49.64 ± 11.72	48.58 ± 10.6	14.03 ± 3.61
Duan et al. (2019) [13]	RAF	26	77.3 ± 9.3	28.6 ± 9.6	9.5 ± 6.8	4.3 ± 1.8
	CFF	23	79 ± 9.8	46.7 ± 8.5	41.3 ± 12.4	18.1 ± 7.2
He et al. (2019) [14]	RAF	30	NA	NR	NR	0.01
	CFF	30	NA	NR	NR	2.39
Wan et al. (2021) [15]	RAF	21	64.12 ± 10.86	12.2 ± 2.11	74.51 ± 7.48	5.52
	CFF	21	88.29 ± 14.29	19.86 ± 3.29	76.92 ± 8.29	10.71
Wang et al. (2019) [16]	RAF	63	65.70 ± 9.87	13.67 ± 4.39	15.25 ± 6.21	9.95 ± 3.72
	CFF	65	73.74 ± 9.78	17.09 ± 4.02	25.511 ± 6.97	13.78 ± 4.39
Zhu et al. (2021) [17]	RAF	50	83.3 ± 31.2	40.1 ± 28.5	11.3 ± 7.3	NR
	CFF	83	44.1 ± 14.8	38.6 ± 21	51.6 ± 40.4	NR

**Figure 2:**
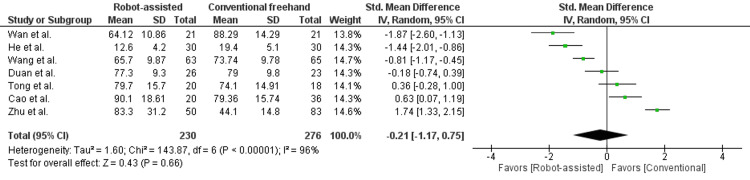
Forest plot of robot-assisted versus conventional freehand femoral neck fixation - operation duration (minutes).

In Figure 2, operation duration was reported in seven studies, enrolling 506 patients. There was no statistically significant difference seen in the mean difference analyses, showing a similar operative duration in both intervention and control groups (MD = -0.21, 95% CI = -1.17 to 0.75, P = 0.66). A high level of heterogeneity was found among the studies (I2 = 96%, P < 0.00001). He et al. reported operation duration as time for cannulated screw insertion rather than total operation duration, but the method was standardized in the conventional freehand group within the same study [14].

**Figure 3:**
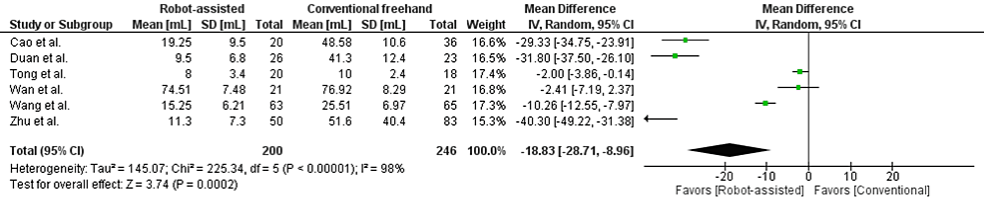
Forest plot of robot-assisted versus conventional freehand femoral neck fixation - intraoperative bleeding (mL).

In Figure 3, intraoperative bleeding was reported in six studies, enrolling 446 patients. There was a statistically significant difference seen in the mean difference analyses, showing less intraoperative bleeding in the robot group (MD = -18.83, 96% CI = -28.71, -8.96, P = 0.0002). A high level of heterogeneity was found among the studies (I2 = 98%, P < 0.00001). Intraoperative bleeding was not reported in He et al. [14].

**Figure 4:**
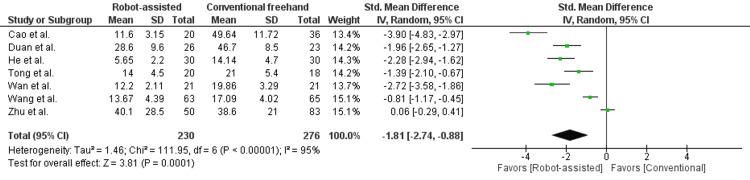
Forest plot of robot-assisted versus conventional freehand femoral neck fixation - intraoperative fluoroscopy exposure.

In Figure 4, the frequency of fluoroscopy was reported in seven studies, enrolling 506 patients. There was a statistically significant difference seen in the mean difference analyses, showing reduced exposure to fluoroscopy in the robot-assisted group compared to the conventional group (MD = -1.81, 95% CI = -2.74 to -0.88, P = 0.0001). A high level of heterogeneity was found among the studies (I2 = 95%, P < 0.00001). He et al. reported fluoroscopy exposure in terms of time during guide pin insertion (seconds), which was accounted for using the standardized MD [14].

**Figure 5:**
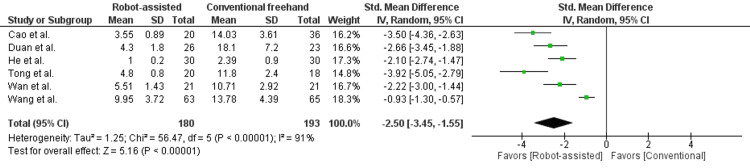
Forest plot of robot-assisted versus conventional freehand femoral neck fixation - frequency of intraoperative drilling.

In Figure 5, the frequency of intraoperative drilling was reported in 6 studies enrolling 373 patients. There was a statistically significant difference seen in the mean difference analyses favoring the robot-assisted group (MD = -7.35, CI = -11.57 to -3.12, P = 0.0007). A high level of heterogeneity was found among the studies (I2 = 91%, P < 0.00001).

Secondary Outcomes

There were three distinct categories of secondary outcomes. Healing and recovery included the Harris score and fracture healing time and rate. Screw placement accuracy included AP and lateral parallelism and neck-width coverage, while the postoperative category included complications such as avascular necrosis and postoperative hospital stay.

**Figure 6:**
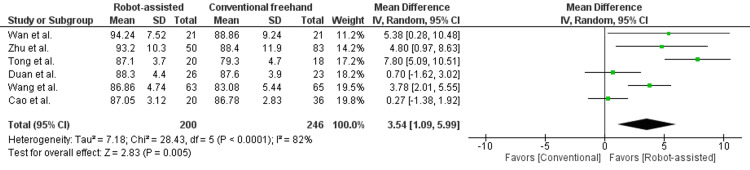
Forest plot of robot-assisted versus conventional freehand femoral neck fixation - Harris scores.

Healing and recovery: In Figure 6, the Harris score was reported in six studies, enrolling 446 patients. There was a statistically significant difference seen in the mean difference analyses, favoring the robot-assisted group (MD = 3.54, 95% CI = 1.09 to 5.99, P < 0.00001). A medium level of heterogeneity was found among the studies (I2 = 52%, P < 0.0001). He et al. [14] reported no significant differences in Harris scores between the two groups. Due to absent SDs, this study was not included in the quantitative analysis.

**Figure 7:**
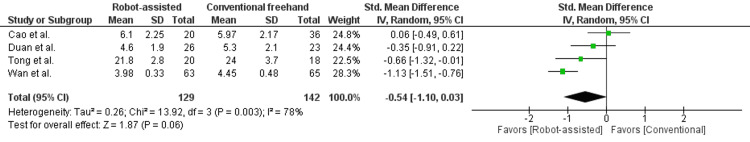
Forest plot of robot-assisted versus conventional freehand femoral neck fixation - fracture healing time (months).

In Figure 7, the fracture healing time was reported in four studies, enrolling 271 patients. There was a statistically significant difference seen in the mean difference analyses, favoring the robot-assisted group (MD= -0.54, 95% CI = -1.10 to -0.03, P = 0.06). A high level of heterogeneity was found among the studies (I2 = 72%, P = 0.003).

**Figure 8:**
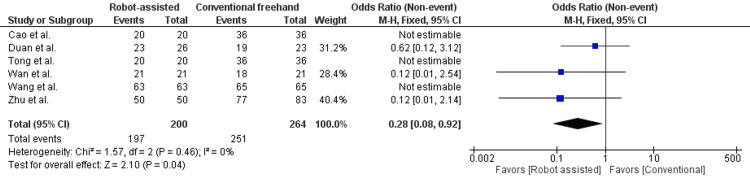
Forest plot of robot-assisted versus conventional freehand femoral neck fixation - fracture healing rate (%).

In Figure 8, the fracture healing rate was reported in seven studies, enrolling 524 patients. There was a statistically significant difference seen in the odds ratio analyses, favoring the robot-assisted group (OR = 0.28, 95% CI = 0.09 to 0.87, P = 0.03). A low level of heterogeneity was found among the studies (I2 = 0%, P = 0.67). The odds ratio was not estimable for Wang et al. [16] as there was 100% healing in both groups and was not included in the plot.

**Figure 9:**
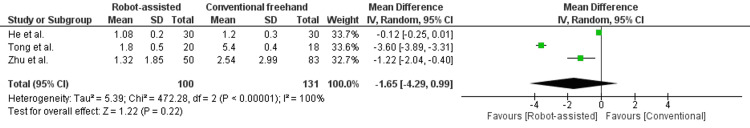
Forest plot of robot-assisted versus conventional freehand femoral neck fixation - anterior-posterior screw placement accuracy.

**Figure 10:**
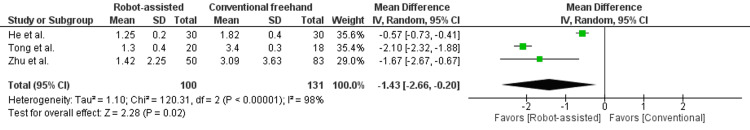
Forest plot of robot-assisted versus conventional freehand femoral neck fixation - lateral screw placement accuracy.

Screw placement accuracy:* *This was assessed in the AP direction and also laterally as seen in Figure 9 and Figure 10. Table 3 summarizes outcomes with regard to screw placement accuracy. Patients in the robotic-assisted fixation group experienced a significant improvement in the functional scoring system, reported as an increase in the parallelism score by Duan et al. [13]. Neck-width coverage, which has been reported in Duan et al. [13], is significantly greater in the robot-assisted group.

**Table 3:** Screw placement accuracy outcomes. NR: not reported

Author and year	Anterior-posterior radiograph parallelism		Lateral radiograph parallelism		Parallelism functional score system		Neck-width area coverage	
	Value (°)	P-value	Value (°)	P-value	Value (points)	P-value	Area (mm^2^)	P-value
Tong et al. (2016) [11]	1.8 ± 0.5 versus 5.4 ± 0.4	NR	1.3 ± 0.4 versus 3.4 ± 0.3	NR	NR	NR	NR	NR
Cao et al. (2017) [12]	NR	NR	NR	NR	NR	NR	NR	NR
He et al. (2019) [14]	1.08 ± 0.2 versus 1.2 ± 0.3	0.437	1.25 ± 0.2 versus 1.82 ± 0.4	0.028	NR	NR	NR	NR
Duan et al. (2019) [13]	NR	NR	NR	NR	24.0 ± 0.6 versus 21.5 ± 1.2	<0.001	72.0 ± 6.7 versus 53.8 ± 10.4	<0.001
Wang et al. (2019) [16]	NR	NR	NR	NR	NA	NR	NR	NR
Wan et al. (2021) [15]	NR	NR	NR	NR	NR	NR	NR	NR
Zhu et al. (2021) [17]	1.32 ± 1.85 versus 2.54 ± 2.99	0.05	1.42 ± 2.25 versus 3.09 ± 3.63	0.001	NR	NR	NR	NR

In Figure 9, AP screw placement accuracy was reported in three studies, enrolling 231 patients. No statistically significant difference was seen between the robot and conventional groups (MD = -1.65, 95% CI = -4.29 to -0.99, P = 0.22). A high level of heterogeneity was found among the studies (I2 = 100%, P < 0.0001).

In Figure 10, lateral screw placement accuracy was reported in three studies, enrolling 231 patients. A statistically significant difference was seen, favoring the robot-assisted group (MD = -1.43, 95% CI = -2.66 to -0.20, P = 0.02). A high level of heterogeneity was found among the studies (I2 = 98%, P < 0.00001).

Postoperative complications: Postoperative complications have been reported in all five studies and can be seen in Table 4. Overall, robot-assisted fixation is associated with fewer complications. The incidence of avascular necrosis is significantly greater in the conventional group compared to the robot-assisted group in Wang et al. [16], He et al. [14], and Zhu et al. [17]. No significant differences were seen between the groups in other studies. According to Duan et al. [13] and Zhu et al. [17], the robot-assisted group was less likely to have femoral head penetration. No significant differences were seen between the groups in other studies. Except for Wang et al. [16], all studies showed that conventional fluoroscopy-guided treatment increases the likelihood of nonunion compared to the robot-assisted group. Screw fixation loosening has been reported in all studies. In He et al. [14], Wang et al. [16], and Wan et al. [15], the conventional treatment is linked with a greater likelihood of screw fixation loosening. No significant differences were seen between the groups in other studies. He et al. [14] showed that secondary fracture displacement was more likely in the conventional group. No significant differences were seen between the groups in other studies. According to He et al. [14], patients in the robot-assisted group were more likely to have limb length shortening post-surgery. No episodes of infection or neurovascular injury were reported in any of the five studies. Zhu et al. [17] found that patients without comorbidities undergoing robot-assisted femoral neck fracture fixation have a statistically significant shorter postoperative hospital stay.

**Table 4:** Fracture healing and postoperative outcomes including healing rate and time, Harris score, and complications. NR: not reported, RAF: robot-assisted fixation, CFF: conventional freehand fixation, AVN: avascular necrosis, NV: neurovascular

Study	Group	N	Fracture healing (N)	Fracture healing (months) (Mean ±SD)	Harris score (0-100) (mean ± SD)	Femoral head AVN (N)	Femoral head penetration (N)	Nonunion (N)	Screw fixation loosening (N)	Secondary fracture displacement (N)	Limb length shortening (N)	Hospital stay (days) (mean± SD)	NV injury (N)	Infection (N)
Tong et al. (2016) [11]	RAF	20	20	21.8 ± 2.8	87.1 ± 4.7	NR	NR	NR	NR	NR	NR	NR	NR	NR
	CFF	20	20	24 ± 3.7	79.3 ± 3.7	NR	NR	NR	NR	NR	NR	NR	NR	NR
Cao et al. (2017) [12]	RAF	36	36	6.1 ± 2.25	87.05 ± 3.12	0	0	0	0	0	0	0	0	0
	CFF	18	16	5.97 ± 2.17	86.78 ± 2.83	0	0	0	0	0	0	0	0	0
Duan et al. (2019) [13]	RAF	26	23	4.6 ± 1.9	88.3 ± 4.4	NR	3	3	0	NR	NR	NR	0	0
	CFF	23	19	5.3 ± 2.1	87.6 ± 3.9	NR	9	4	0	NR	NR	NR	0	0
He et al. (2019) [14]	RAF	30	30	NR	85.2	1	5	0	0	0	5	NR	NR	NR
	CFF	30	29	NR	83.45	3	5	1	1	2	2	NR	NR	NR
Wan et al. (2021) [15]	RAF	21	21	3.98 ± 0.33	94.24 ± 7.52	0	NR	0	0	0	NR	NR	NR	0
	CFF	21	18	4.45 ± 0.48	88.86 ± 9.24	0	NR	3	3	0	NR	NR	NR	0
Wang et al. (2019) [16]	RAF	63	63	NR	86.86 ± 4.74	0	NR	0	0	0	NR	NR	NR	0
	CFF	65	65	NR	83.08 ± 5.44	1	NR	0	2	0	NR	NR	NR	0
Zhu et al. (2021) [17]	RAF	50	50	NR	93.2 ± 10.3	3	0	0	0	0	NR	8.6 ± 2.8	0	0
	CFF	83	76	NR	88.4 ± 11.9	20	2	6	0	0	NR	11.1 ± 3.4	0	0

Methodological Quality and Risk of Bias Assessment 

As seen in Table 5, the Cochrane Collaboration tool was utilized to assess the quality of the randomized studies. Overall, both studies have a low risk of bias in most areas; however, due to a lack of information in the study methodologies, selection bias and performance bias were rated as unclear for both groups. Detection bias was also unclear for Wan et al. [15].

**Table 5:** Assessment of the risk of bias of the randomized trials using the Cochrane Collaboration tool.

Author and year	Bias	Authors’ judgment	Support for judgment
Wan et al. (2021) [15]	Random sequence generation (selection bias)	Low risk (green)	42 patients were randomized into two equal groups of 21 patients
	Allocation concealment (selection bias)	Unclear risk of bias (yellow)	No information given
	Blinding of participants and personnel (performance bias)	Unclear risk of bias (yellow)	No information given
	Blinding of outcome assessment (detection bias)	Unclear risk of bias	No information given
	Incomplete outcome data (attrition bias)	Low risk	No patient dropout
	Selective reporting (reporting bias)	Low risk	No reporting bias was evidenced
	Other bias	Low risk	Clear study protocol, no other bias detected
He et al. (2019) [14]	Random sequence generation (selection bias)	Low risk	60 patients were randomized into two equal groups of 30 patients
	Allocation concealment (selection bias)	Unclear risk	No information given
	Blinding of participants and personnel (performance bias)	Unclear risk	No information given
	Blinding of outcome assessment (detection bias)	Low risk	No information given
	Incomplete outcome data (attrition bias)	Low risk	No patient dropout
	Selective reporting (reporting bias)	Low risk	No reporting bias was found
	Other bias	Low risk	Clear study protocol, no other bias detected

The quality of the nonrandomized studies was assessed using the Newcastle-Ottawa Scale (NOS) [18] (Table 6), which uses a star system to analyze selection, comparability, and outcome. The selection, comparability, and outcome domains have a maximum star score of four, two, and three stars, respectively. Overall, all three studies were of high quality based on the Agency for Healthcare Research and Quality (AHRQ) standards [19]. All five nonrandomized studies (Tong et al., Cao et al., Duan et al, Wang et al., and Zhu et al.) [11,12,13,16,17] have shown a high quality of patient selection with clear inclusion and exclusion criteria. This is represented by a score of four stars (Table 6). Patients who underwent both robot-assisted fixation and conventional freehand surgery were derived from the same surgical records. Both groups did not have the outcome of interest at the start of the study. The study compares the effectiveness of robot-guided surgery to manual fixation in the control group, exhibiting clear comparability. Several patient factors such as age, fracture subtype, and causes were standardized and controlled. All patients continued until the end of follow-up, avoiding attrition bias. The length of follow-up was adequate but not enough in the studies and was required to be longer to assess efficacy outcomes such as healing rate and long-term complications such as AVN.

**Table 6:** Newcastle-Ottawa Scale (NOS) to assess the quality of nonrandomized studies.

Study	Selection	Comparability	Outcome
Tong et al. [11]	***	**	***
Cao et al. [12]	***	**	***
Duan et al. [13]	****	**	***
Wang et al. [16]	****	**	***
Zhu et al. [17]	****	**	***

Discussion

In a pooled analysis of seven trials [11-17], robotic-assisted surgery was associated with a decrease in intraoperative bleeding loss [11,12,13,15,16,17], due to the lower number of holes drilled and hence less trauma as reported in both Duan et al. [13] and Wan et al. [15]. The frequency of intraoperative fluoroscopy use was significantly reduced in the robotic-assisted surgery arm, as shown in the studies Tong et al. [11], Cao et al. [12], Duan et al. [13], He et al. [14], Wan et al. [15], and Wang et al. [16]. This results in less radiation exposure and greater safety for both patients and surgical staff [13]. The robot-assisted device had no overall effect on the operation duration, despite being significantly lower in studies by Wan et al. [15] and Wang et al. [16], and slightly shorter as reported by Duan et al. [13]. However, the operation duration was significantly increased in the study conducted by Zhu et al. [17] due to the additional surgical steps associated with the new robotic system, as well as the surgeons’ lack of expertise in dealing with the manipulator in the early period [8,15]. The operation duration extended from approximately 60 to 90 minutes in all studies, but the mean time taken in the conventional group in one trial [17] was much shorter compared to others and can potentially be due to varying skill levels between surgeons, lower case complexity, and less intraoperative complications. Future studies are needed, with adequate training and more staff to perform the planning steps [20] and the inclusion of larger sample sizes to accurately evaluate the effect on the operation duration, as it stays undetermined.

In terms of postoperative outcomes, the Harris hip score, which can be used as a prognostic scale [13,15,16,17], showed significantly higher outcomes in robot-assisted fixation in four of the studies; the higher the score, the better the outcome [21]. The main factors affecting prognosis postoperatively include fracture classification by both Pauwel and Garden classifications and age [17]. Nonunion and AVN complications are rare in patients with Garden type I and II compared to type III and IV [17]. Similar results were yielded according to the Pauwel classification [14]. Regardless of the age or fracture subtype, robot navigation surgery was associated with a lower incidence of nonunion [12,13,17] and AVN [14,16,17], as suggested by three studies [14,16,17]. AVN complications can arise from multiple or inaccurate guide needle insertion [1] or fixating screw [14,17], where it penetrates the femoral head and blood supply [1]. Guidewire insertion frequency was lower and more accurately positioned in the robot-assisted surgery group, as shown in two studies [13,16]. This was due to the device having a correction function, carrying out adjustments in the case of guide needle deviation, hence improving safety. As a result, screw parallelism when arranged in an “inverted angle” effect was significantly improved [13,14,17], as well as the neck-width coverage, due to the maximal spread of the three parallel screws [19,20] was significantly enlarged. Clinically, this has been shown to enhance the stability of fracture fixation [13,22,23], which is important for fracture compression and healing [24]. Additionally, the robot-guided surgery resulted in a reduction in the healing time [13,15], as well as better outcomes in the healing rate, accounting for more patients healing completely [13,15,17]. Postoperative healing time was related to drilling frequency [5]. With the frequency being lower when utilizing robotic guidance, the healing time and rate results are consistent with what has been shown in both studies in terms of drilling frequency [13,15]. Moreover, no cases of wound infection and vascular or nerve damage were reported, exhibiting a high level of safety for patients and hence avoiding severe adverse effects that can have debilitating consequences on their quality of life [11-17]. Furthermore, more cases of screw loosening or dislocation occurred in the conventional freehand surgery group, which was potentially attributed to the lower accuracy and higher frequency of screw insertion [14-16]. Finally, two cases of secondary fracture displacement occurred in the conventional surgery group compared to none in the robotic-assisted group, as reported by He et al. [14]; however, it was not a considerable complication in the other studies. This can further emphasize the greater level of accuracy and postoperative stability offered by the robotic assistance, resulting in fewer complications such as malunion, fracture relapses, and potential avascular necrosis as outlined in our studies [11-17].

Currently, robotic-assisted surgery is becoming more widely used in many orthopedic surgeries [9,25,26] and has shown superior results when compared to freehand surgery. Better outcomes with regard to reduced radiation exposure, greater healing rates, and potentially reduced hospitalization stay, as well as improved postoperative stability and mobilization, would support the theory of considering robotic-guided surgery as gold standard management in future practice, despite being a more costly option [13]. Additionally, manipulator’s complications were far less common [17]. However, repeated practice is required as it is believed to reduce the operation time [11-17]. This can have an impact on the efficiency, reducing the waiting lists and increasing bed availability, due to better recovery rate and hence faster discharge. This device can reduce the costs related to complications and longer hospital stays and be deemed cost-effective.

A strength of the studies included is having a good range of defined outcomes that are significant, clearly favoring the new invention. Additionally, effective selection of the cohorts was derived from secure surgical records in the nonrandomized studies, choosing patients who are representative of both groups being compared. Some variables, such as age, fracture side, subtype, and causes, were standardized (no significant difference) to avoid confounding bias. Follow-up of all patients continued until the end, avoiding attrition bias. Limitations included a short follow-up period in the studies as complications such as AVN usually occur 2-3 years after NOF [23]. This can also result in inaccuracies when determining the healing rate, at which patients healed completely, as it can take time. Studies with a shorter follow-up period would show less significance. Additionally, all studies were single-center, and five [11,12,13,16,17] were not randomized. Sample sizes were small as well. For improvement, further multicenter randomized controlled trials with larger sample sizes and follow-up periods are recommended. Moreover, some outcomes were only evaluated by two studies, hence being underpowered, and the units reported were sometimes not standardized, making it harder to combine the results occasionally.

## Conclusions

Despite being still under development, robot-assisted surgery has so far proven to be a more effective technique compared to the manual surgical technique, as displayed by the promising results of our meta-analysis. Intraoperative blood loss and drilling and fluoroscopy frequency were significantly reduced in the robot-assisted group; however, there was no significant difference in operation duration between robot-assisted and conventional screw fixation. Robot-assisted fixation is surgically more accurate and has better postoperative Harris scores and healing rates, although there was no significant difference in terms of healing times. Postoperative complications, including avascular necrosis and nonunion, are reduced in the robot-assisted group. Improved patient safety and quality of life were evidenced, further emphasizing the benefit this device can have on the healthcare system. More randomized controlled trials that have higher-quality evidence are required in the future to evaluate the outcomes that have been undetermined in this analysis. This would have allowed adequate training for surgeons after a longer implementation of the robotic systems in the healthcare system, providing a better insight into their effectiveness.

## References

[1] Kazley J, Kaushik B (2021). Femoral neck fractures. https://www.ncbi.nlm.nih.gov/books/NBK537347/.

[2] Johnell O, Kanis JA (2006). An estimate of the worldwide prevalence and disability associated with osteoporotic fractures. Osteoporos Int.

[3] Rogmark C, Kristensen MT, Viberg B, Rönnquist SS, Overgaard S, Palm H (2018). Hip fractures in the non-elderly-who, why and whither?. Injury.

[4] Protzman RR, Burkhalter WE (1976). Femoral-neck fractures in young adults. J Bone Joint Surg Am.

[5] Browbank I, Bouazza-Marouf K, Schnabler J (2000). Robotic-assisted internal fixation of hip fractures: a fluoroscopy-based intraoperative registration technique. Proc Inst Mech Eng H.

[6] Liebergall M, Ben-David D, Weil Y, Peyser A, Mosheiff R (2006). Computerized navigation for the internal fixation of femoral neck fractures. J Bone Joint Surg Am.

[7] Wang JQ, Wang Y, Feng Y (2017). Percutaneous sacroiliac screw placement: a prospective randomized comparison of robot-assisted navigation procedures with a conventional technique. Chin Med J (Engl).

[8] Peng YN, Tsai LC, Hsu HC, Kao CH (2020). Accuracy of robot-assisted versus conventional freehand pedicle screw placement in spine surgery: a systematic review and meta-analysis of randomized controlled trials. Ann Transl Med.

[9] Al-Naseem A, Sallam A, Gonnah A, Masoud O, Abd-El-Barr MM, Aleem IS (2021). Robot-assisted versus conventional percutaneous sacroiliac screw fixation for posterior pelvic ring injuries: a systematic review and meta-analysis. Eur J Orthop Surg Traumatol.

[10] Moher D, Liberati A, Tetzlaff J, Altman DG (2009). Preferred reporting items for systematic reviews and meta-analyses: the PRISMA statement. Ann Intern Med.

[11] Tong Y, Luo X, Wu G, Shi W (2016). [Comparative study on fixation with percutaneous cannulated screws assisted by robot navigation and conventional surgery with manual positioning for femoral neck fractures]. Zhongguo Xiu Fu Chong Jian Wai Ke Za Zhi.

[12] Cao Y, Zhao Y, Hu L (2017). [Clinical application of computer-assisted cannulated screw internal fixation system based on error correction method for femoral neck fractures]. Zhongguo Xiu Fu Chong Jian Wai Ke Za Zhi.

[13] Duan SJ, Liu HS, Wu WC, Yang K, Zhang Z, Liu SD (2019). Robot-assisted percutaneous cannulated screw fixation of femoral neck fractures: preliminary clinical results. Orthop Surg.

[14] He M, Han W, Zhao CP, Su YG, Zhou L, Wu XB, Wang JQ (2019). Evaluation of a bi-planar robot navigation system for insertion of cannulated screws in femoral neck fractures. Orthop Surg.

[15] Wan L, Zhang X, Wu D (2021). Application of robot positioning for cannulated screw internal fixation in the treatment of femoral neck fracture: retrospective study. JMIR Med Inform.

[16] Wang XD, Lan H, Li KN (2019). Treatment of femoral neck fractures with cannulated screw invasive internal fixation assisted by orthopaedic surgery robot positioning system. Orthop Surg.

[17] Zhu ZD, Xiao CW, Tan B (2021). Tirobot-assisted percutaneous cannulated screw fixation in the treatment of femoral neck fractures: a minimum 2-year follow-up of 50 patients. Orthop Surg.

[18] Stang A (2010). Critical evaluation of the Newcastle-Ottawa scale for the assessment of the quality of nonrandomized studies in meta-analyses. Eur J Epidemiol.

[19] Farquhar M (2008). AHRQ quality indicators. Patient safety and quality: an evidence-based handbook for nurses.

[20] Müller MC, Belei P, Pennekamp PH, Kabir K, Wirtz DC, Burger C, Weber O (2012). Three-dimensional computer-assisted navigation for the placement of cannulated hip screws. A pilot study. Int Orthop.

[21] Nilsdotter A, Bremander A (2011). Measures of hip function and symptoms: Harris Hip Score (HHS), Hip Disability and Osteoarthritis Outcome Score (HOOS), Oxford Hip Score (OHS), Lequesne Index of Severity for Osteoarthritis of the Hip (LISOH), and American Academy of Orthopedic Surgeons (AAOS) Hip and Knee Questionnaire. Arthritis Care Res (Hoboken).

[22] Selvan VT, Oakley MJ, Rangan A, Al-Lami MK (2004). Optimum configuration of cannulated hip screws for the fixation of intracapsular hip fractures: a biomechanical study. Injury.

[23] Gurusamy K, Parker MJ, Rowlands TK (2005). The complications of displaced intracapsular fractures of the hip: the effect of screw positioning and angulation on fracture healing. J Bone Joint Surg Br.

[24] Leighton RK (2006). Fractures of the neck of the femur. Fractures in adults, sixth edition.

[25] Liu HS, Duan SJ, Xin FZ, Zhang Z, Wang XG, Liu SD (2019). Robot-assisted minimally-invasive internal fixation of pelvic ring injuries: a single-center experience. Orthop Surg.

[26] Negrín R, Duboy J, Reyes NO (2020). Robotic-assisted unicompartmental knee arthroplasty optimizes joint line restitution better than conventional surgery. J Exp Orthop.

